# Pancreatic SABR using peritumoral fiducials, triggered imaging and breath-hold

**DOI:** 10.3389/pore.2023.1611456

**Published:** 2023-12-21

**Authors:** Katalin Kisivan, Andrea Farkas, Peter Kovacs, Csaba Glavak, Gabor Lukacs, Karoly Mahr, Zsolt Szabo, Melinda Petone Csima, Akos Gulyban, Zoltan Toth, Zsolt Kaposztas, Ferenc Lakosi

**Affiliations:** ^1^ Department of Radiotherapy, Somogy County Kaposi Mór Teaching Hospital, Kaposvár, Hungary; ^2^ Department of Medical Oncology, Somogy County Kaposi Mór Teaching Hospital, Kaposvár, Hungary; ^3^ Department of Medical Oncology, Zala County Szent Raphael Hospital, Zalaegerszeg, Hungary; ^4^ Institute of Education, Hungarian University of Agricultural and Life Sciences, Gödöllő, Hungary; ^5^ Faculty of Health Sciences, University of Pecs, Pecs, Hungary; ^6^ Department of Medical Physics, Institut Jules Bordet, Brussels, Belgium; ^7^ Radiophysics and MRI Physics Laboratory, Université Libre De Bruxelles (ULB), Brussels, Belgium; ^8^Medicopus Nonprofit Ltd., Somogy County Kaposi Mór Teaching Hospital, Kaposvár, Hungary; ^9^ PET Center, Somogy County Kaposi Mór Teaching Hospital, Kaposvár, Hungary; ^10^ Department of Surgery, Somogy County Kaposi Mór Teaching Hospital, Kaposvár, Hungary

**Keywords:** motion control, pancreatic cancer, triggered imaging, stereotactic ablative radiotherapy, deep inspiration breath hold

## Abstract

**Background:** We aim to present our linear accelerator-based workflow for pancreatic stereotactic ablative radiotherapy (SABR) in order to address the following issues: intrafractional organ motion management, Cone Beam CT (CBCT) image quality, residual errors with dosimetric consequences, treatment time, and clinical results.

**Methods:** Between 2016 and 2021, 14 patients with locally advanced pancreatic cancer were treated with induction chemotherapy and SABR using volumetric modulated arc therapy (VMAT). Internal target volume (ITV) concept (5), phase-gated (4), or breath hold (5) techniques were used. Treatment was verified by CBCT before and after irradiation, while tumor motion was monitored and controlled by kV triggered imaging and beam hold using peritumoral surgical clips. Beam interruptions and treatment time were recorded. The CBCT image quality was scored and supplemented by an agreement analysis (Krippendorff’s-α) of breath-hold CBCT images to determine the position of OARs relative to the planning risk volumes (PRV). Residual errors and their dosimetry impact were also calculated. Progression free (PFS) and overall survival (OS) were assessed by the Kaplan-Meier analysis with acute and late toxicity reporting (CTCAEv4).

**Results:** On average, beams were interrupted once (range: 0–3) per treatment session on triggered imaging. The total median treatment time was 16.7 ± 10.8 min, significantly less for breath-hold vs. phase-gated sessions (18.8 ± 6.2 vs. 26.5 ± 13.4, *p* < 0.001). The best image quality was achieved by breath hold CBCT. The Krippendorff’s-α test showed a strong agreement among five radiation therapists (mean K-α value: 0.8 (97.5%). The mean residual errors were <0.2 cm in each direction resulting in an average difference of <2% in dosimetry for OAR and target volume. Two patients received offline adaptation. The median OS/PFS after induction chemotherapy and SABR was 20/12 months and 15/8 months. No Gr. ≥2 acute/late RT-related toxicity was noted.

**Conclusion:** Linear accelerator based pancreatic SABR with the combination of CBCT and triggered imaging + beam hold is feasible. Peritumoral fiducials improve utility while breath-hold CBCT provides the best image quality at a reasonable treatment time with offline adaptation possibilities. In well-selected cases, it can be an effective alternative in clinics where CBCT/MRI-guided online adaptive workflow is not available.

## Introduction

Pancreatic cancer (PC) is the seventh leading cause of cancer-related deaths worldwide, with a 5 years survival rate of less than 10% [1–4]. Currently, complete resection is the only curative approach, however only 10%–15% of patients are diagnosed with resectable disease. Gemcitabine or 5-fluorouracil, leucovorin, irinotecan, and oxaliplatin (Folfirinox)-based induction chemotherapy (iCT) followed by radio-chemotherapy (RCT) is the mainstay of treatment for the remaining subset of patients presenting with either locally advanced (LAPC) or borderline resectable disease [1, 2]. Dose escalated RT/RCT, or stereotactic ablative radiotherapy (SABR), have emerged as effective treatment options in recent years. Prospective and retrospective data showed an impressive median overall survival of 13.9–26 months with an acceptable ≥ Grade (Gr.) 3 morbidity of 6%–12.8% [5–15].

Delivery of high doses is challenging during LAPC-SABR due to the close vicinity of organs at risk (OARs) (duodenum, gastric wall, small/large bowels) with their large intra- and interfractional positional and volumetric changes. This could potentially increase the dose to these organs and may be responsible for serious late toxicity [6, 16, 17]. Hence, both the visualization of OARs and intrafraction organ motion management are cornerstones during LAPC-SABR. MRI linear accelerator (Linac), with its superior soft tissue contrast, in conjunction with daily adaptation and real-time organ motion management has great potential [14], however, it is not yet widely available. Alternatively, CT-on rail [12] or CBCT based adaptive workflows (Ethos, Varian Medical Systems, Palo Alto, CA, United States) could be used for similar purposes [18]. Still, one should explore and address the above-mentioned needs on conventional Linacs equipped with cone beam CT (CBCT) where no adaptive workflows are available.

The image quality of abdominal CBCT on Linacs—especially in 4D mode—is suboptimal due to motion and gas artefacts, necessitating the use of fiducials and a better selection of CBCT acquisitions [19, 20]. Fiducials also form the backbone of any intrafraction X-ray based image-guided radiation therapy (IGRT). Most commonly, intra-tumoral fiducials are used. However, they have the disadvantage of not providing any spatial information or landmarks for the positions of the target and OARs. Time efficiency is also a crucial factor since advanced techniques such as gating or tracking are prone to have longer treatment times and consequently more room for errors.

Our goal was to find the most appropriate and time efficient IGRT workflow on our TrueBeam linear accelerator (Varian Medical Systems, Palo Alto, CA, United States) equipped with the Advanced IGRT & Motion Package, allowing intrafractional kilovoltage triggered imaging and Beam Hold.

This system is able to acquire repetitive 2D kV image images during SABR, autodetect different type of fiducials, and hold the treatment beam if their positions do not meet the pre-defined deviation limit [21]. We investigated the image quality and time efficiency of deep inspiration breath-hold (DIBH) technique as the most promising candidate for achieving improved image quality [22] and, as a consequence, offline adaptation. We also introduced peritumoral fiducials, which could potentially expand the utility of fiducial based IGRT. Finally, we combined these techniques with the whole available spectra of 3D/4D CBCTs acquired before (pre-CBCT), and after (post-CBCT) pancreatic SABR. As far as we are aware, this kind of workflow has never been published before.

In this report our primary aim was to present this IGRT workflow for pancreatic SABR, including motion management strategies, CBCT image quality, and time efficiency, especially focusing on breath-hold technique and its offline adaptive aspects. Secondarily, clinical efficacy was also demonstrated.

## Methods and materials

Between 2016 and 2021, fifteen patients with primary or recurrent LAPC were treated with volumetric-modulated arc therapy (VMAT) as a five-fraction SABR or hypofractionated dose-escalated RT (DE-RT). Patients with large (>6 cm) tumors with infiltration of luminal structures and/or were unresponsive to iCT with persistently elevated post-CT carbohydrate antigen (CA19-9) (>300 IU/mL) or extensive nodal disease were not eligible and directed to conventional RCT with capecitabine or systemic treatment. Patients were treated based on the approval of the Central Ethics Committee (OGYÉI/5301/2018). Finally, 14/15 patients were analyzed since one patient refused neoadjuvant chemotherapy and progressed only 3 months after SABR.

**FIGURE 1:**
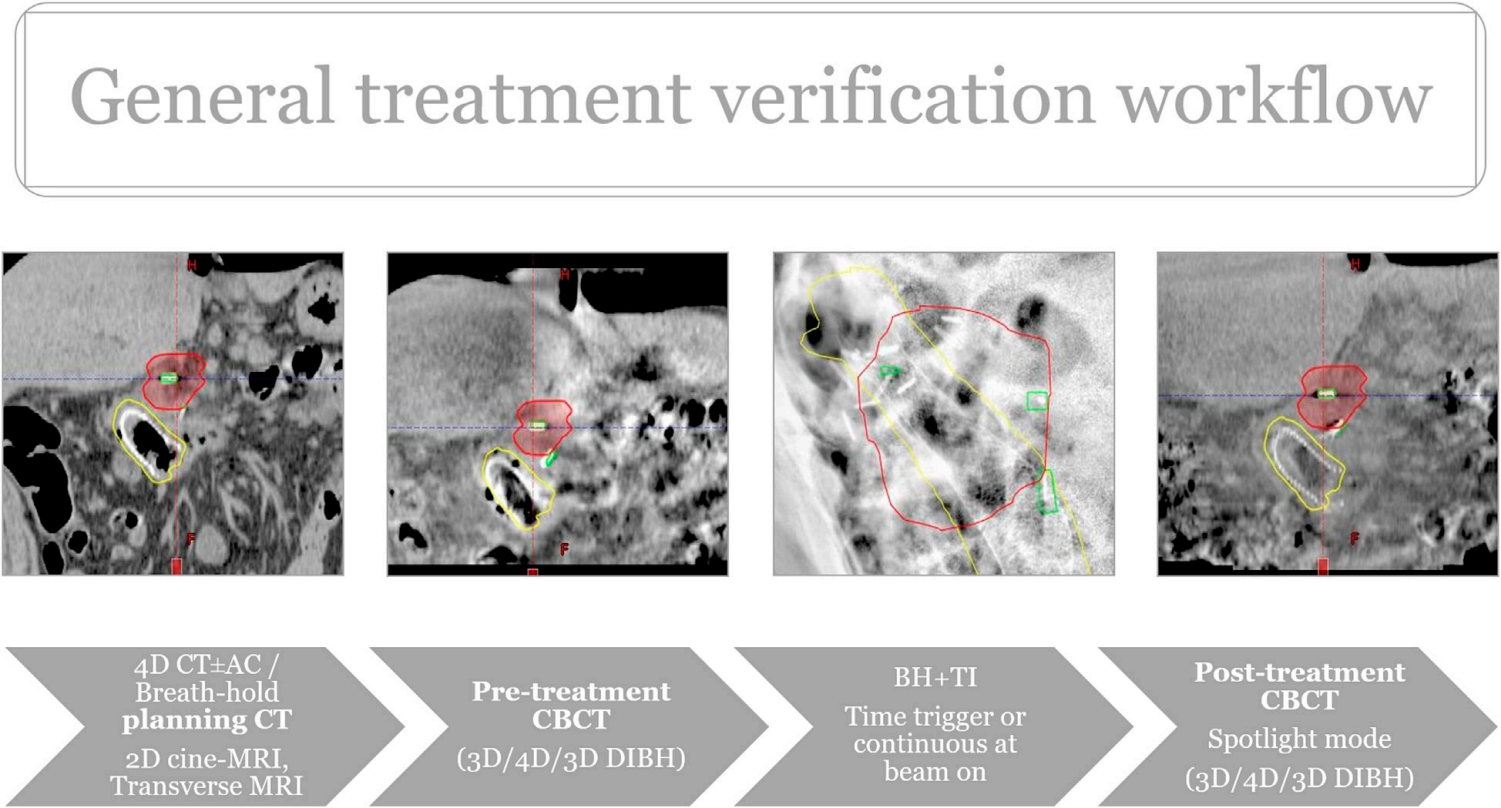
Verification workflow.

The workflow is characterized by the use of fiducials followed by multimodal imaging in the supine position for contouring and planning purposes. A minimum of two types of imaging at two different time points were acquired to obtain relevant information on movement patterns and filling status of OARs. This is the most reliable way of creating internal target volumes (ITV) and planning Organs at risk volumes (PRV_OARs) to select the most appropriate verification protocol for the patient: ITV-based RT with abdominal compression +TI, Phase-gated RT with abdominal compression + triggered imaging or breath-hold RT+ triggered imaging. Pretreatment imaging may include cine MRIs (Siemens Biograph mMRI, 3T, TR 351 ms, TE 1 ms, Flip Angle (FA) 8°, field of view (FOV) 350 mm, Slice thickness/spacing 20/0 mm, one slice, 50 measures, 18 s) in sagittal and coronal planes completed with T2-weighted axial images in the treatment planning position. Initial diagnostic scans were also co-registered with the planning CT (Figure 1).

**FIGURE 2:**
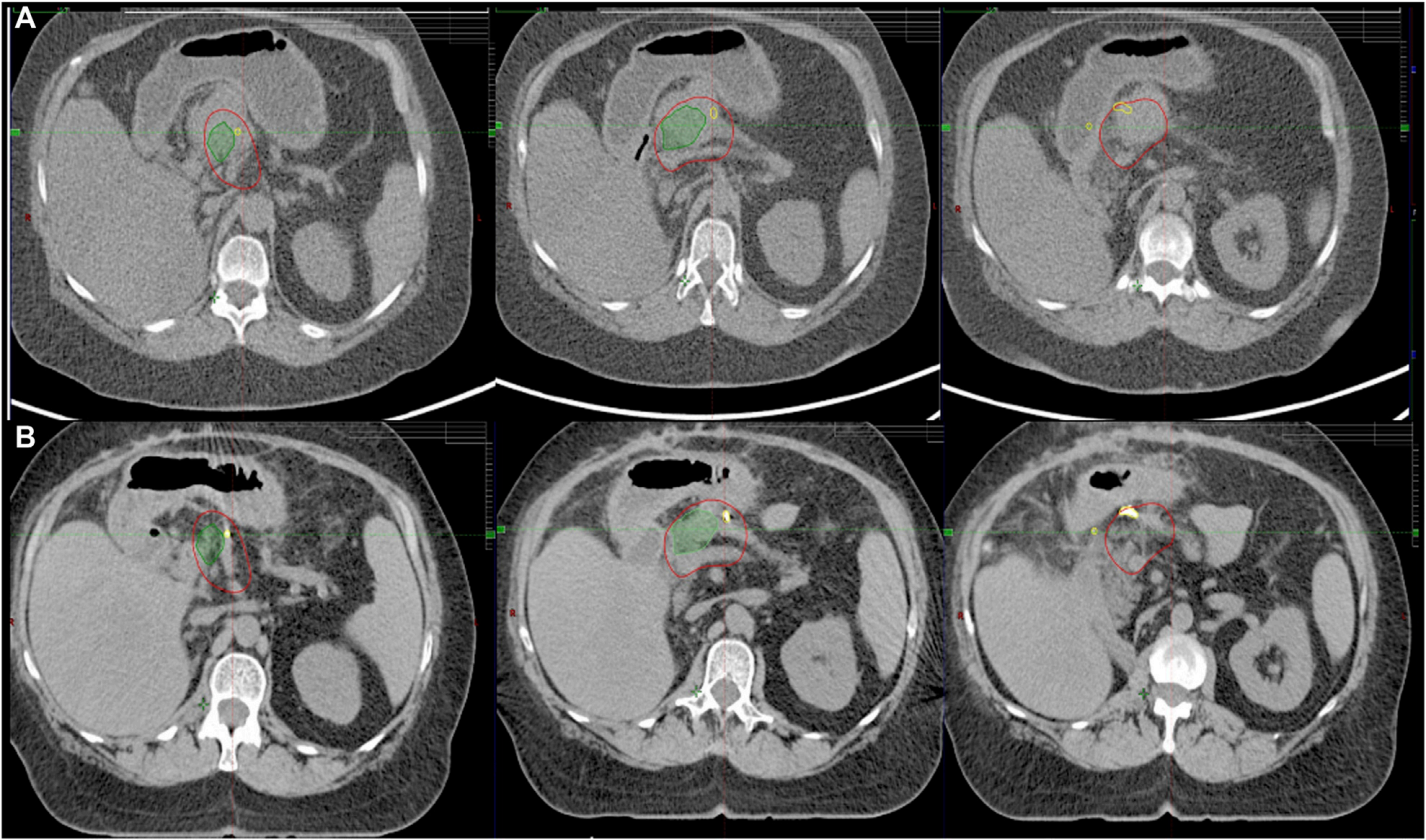
Corresponding axial CT slices on diagnostic scan **(A)** and treatment planning CT **(B)** fused by rigid registration. LAPC with abutment of gastric wall. Residual tumor (green), CTV (red). Note the implanted titanium clips (yellow) between the target volume and OARs assisting in target volume delineation.

**FIGURE 3:**
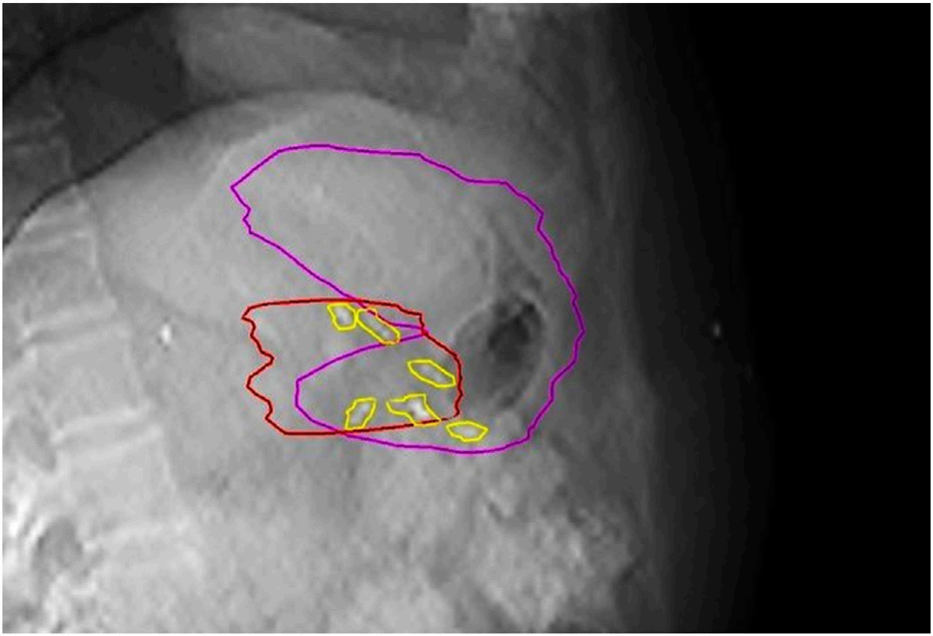
Multiple peritumoral fiducials (yellow) provide multidimensional visualization of the tumor (red) on triggered imaging, while certain clips indirectly represent the preserved gastric wall (purple).

Different types of fiducials were used: lipiodol, titanium clips, bile duct stent. Titanium clips were either already present in the postoperative bed (in case of local relapses) or placed around the tumor at the OARs interfaces during a surgical exploration. The latter provides a proper target volume delineation on CT and allows for multidimensional visualization on TI. It also helps recognize positional and volumetric changes of OARs (Figure 2). While functioning as an indirect surrogate for OARs on intrafractional kV imaging (Figure 3). Lipiodol was injected into the tumor via a transabdominal route. In a minority of the cases a surrogate structure, such as a biliary stent, was used for daily image guidance. Proton -pump inhibitors, antiemetics, and prokinetics were also used as part of the treatment protocol. Patients were simulated in the supine position with arms up after a minimum of 2–3 h fasting. 3D/4D CT scan with abdominal compression [thermoplastic mask with underneath Styrofoam block, from 2019 Zfix (Qfix, Avondale, PA, United States)] or breath-hold CT was performed with Real-time Position Management (RPM). Candidates for breath-hold had a training session at the Linac before simulation to evaluate patient suitability and achievable and reproducible breath-hold levels. Minimum eligibility criteria required the patients to be able to hold their breaths for a minimum of 20 s. During breath-hold-SABR only audio-coaching was available.

Target definition followed international recommendations [23, 24]. The GTVp was defined on a tri-phase contrast-CT. Any major vessel within 5 mm of the tumor had its full circumference included. Fibrotic areas near vessels were also included in the GTVp. Initial diagnostic scans were also co-registered and together with the fiducials helped to finalize GTVp (Figure 2). ITV was created by using motion information from breath hold scans and/or 4D-CT. An ITV-to-PTV margin of 5 mm was applied to generate the PTV. A high dose area within PTV consisted of subtracting the gastrointestinal PRV from the GTVp without margin. GI PRV represents all positional/volumetric changes on different scans with a 3–5 mm expansion. GTVn was also defined as present. Elective nodes (elective nodal irradiation, ENI) were included if nodal disease was present after ICT.

SABR was delivered in five daily fractions for 1.5–2 weeks, prescribing 33 Gy (BED_10_:55 Gy) to the PTV. At the beginning of our learning curve, a homogeneous dose prescription was applied (N:9). With further experience, a dose escalation was carried out to the high-dose area with SIB approach up to 40 Gy (33/40Gy BED_10_:55/72 Gy, N:4). The prescribed dose was reduced to 25 (D99 > 25 Gy) on the overlap area between the PTV and PRV if necessary. In one patient with extensive nodal disease even after iCT, a hypofractionated regimen was applied, consisting of 15 consecutive fractions up to a total dose of 37.5 (elective node)/52.5/60 Gy (macroscopic tumor, BED_10_: 84 Gy). Initially, the normal tissue constraints followed those used in the Herman trial [7], and thereafter followed the recommendation of the UK SABR consortium guideline and published American recommendations [23, 24]. Treatment plans were designed and optimized according to two 6-MV full volumetric modulated arcs (VMAT; TrueBeam v2.7) using flattening filter free (1400 MU/min) photon beams.

**FIGURE 4:**
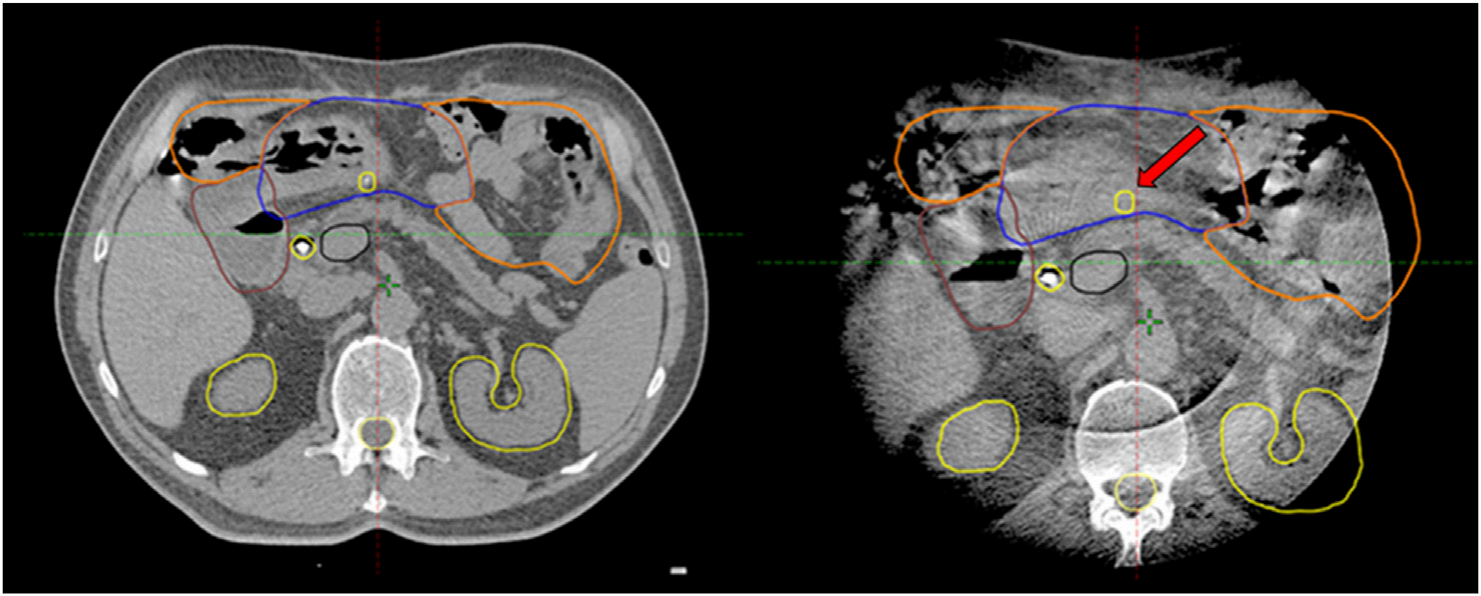
Corresponding axial slices on planning breath-hold-CT and breath-hold-CBCT (half fan mode): Systematic posterior displacement of the stomach (blue) and clip (arrow) was observed during the 1–4th fractions becoming closer to the high-dose area. Consequences/conclusions: 1. The position of this clip (arrow) is no longer relevant during intrafractional triggered imaging 2. This simultaneously alerts radiation therapists to the anatomical changes on CBCT.

Treatment verification started with 3D/4D CBCTs (3D Thorax mode, Slice thickness: 2 mm, 125 kVp and 270 mAs, 4D Thorax mode, Slice thickness: 2 mm, 125 kVp and 672 mAs) followed by triggered imaging and Beam Hold. In the case of breath, breath-hold-CBCT was acquired. Matching was based on fiducials with OAR verification at the same time. During treatment the radiation therapists (RTT) were responsible for manual beam hold (passive beam hold). Time triggering with 3s frequency was applied continuously or when the beam was on (Supplementary Video S1). Overlay structures served as the deviation limit for triggered imaging: 1) breath-hold: Fiducials plus 3 mm, 2) ITV- or Phase-gated SABR: summation of clips’ contours from each or selected breathing phases. In the case of deviation limit violation, treatment delivery was manually interrupted and corrections using additional imaging (2D/3D match ± CBCT), or an adjustment of the gating threshold, was performed. The post-CBCT consisted of a faster half fan mode (Spotlight mode, Slice thickness: 2 mm, 125 kVp and 750 mAs, time 33 s) (Figure 4). In two breath-hold cases OARs were systematically delineated on each pre-and post-CBCTs with offline dose prediction per fraction. If dose constraint per fraction was not fulfilled, a new PRV was generated using CBCT contours and a dose re-optimization was done. If PRVs were violated on pre-CBCT, treatment was prohibited, and the same protocol was applied. Then, patients were treated according to the most appropriate plan based on the actual OARs anatomy (Plan of the day approach). The accumulated D0.5cc for OARs and D98%, D2% for the target was calculated as the mean of each DVH parameter across all CBCT contours in each patient.

Descriptive statistics were given for clinical variables, toxicity, and outcomes.

CBCT image quality for OARs was evaluated on a scale of 1–4 (1: inadequate, 2: doubtful, 3: acceptable, 4: excellent) on full arc pre-CBCT and half fan post CBCTs. Image quality of different CBCT modes (Free breathing, 3D CBCT breath-hold, 4D CBCT AIP, and 4D CBCT MIP) was compared between with Kruskal-Wallis *H* test and Mann-Whitney *U* test with a significance level of *p* < 0.05. Furthermore, we performed a Krippendorff agreement analysis among five radiation therapists (RTT) on 4 LAPC cases retrospectively. They were asked to judge whether the OARs respect their PRV on 40 CBCTs (yes/no). The pre-CBCT was a full arc, while the post-CBCT was a half fan acquisition. The Krippendorff’s-α coefficient (*α* ≥ 0.8) [25] was calculated and compared between the two image acquisition types (McNemar’s test at a significance level of *p* < 0.05).

Treatment time was compared between ITV-, phase gated- and breath-hold-gated SABR with Kruskal-Wallis *H* test and Mann-Whitney *U* test with a significance level of *p* < 0.05. The average times of the workflow were extracted from ARIA: 1) total treatment time (from the start of pre-CBCT to the end of post-CBCT); 2) treatment delivery time (from the start of the first arc to the end of the second arc); 3) “beam on” time + triggering time; and 4) time for 2D/3D match ± other operational time (CBCT analysis, couch movements, settings for Beam Hold, treatment field selection, etc.) Beam interruptions and treatment time were also recorded. Correlation analysis (Spearman) was also performed between the different time spans and number of corrections.

Overall survival (OS), Progression free survival (PFS), and Local progression free survival (LPFS) were calculated and described by the Kaplan-Meier analysis and reported, both from the start of iCT and the end of SABR. OS/PFS/LPFS were defined as the period from the start date of ICT and end date of SABR to the date of any death/progression/local progression. Acute and late toxicity side effects were evaluated using the Common Toxicity Criteria for Adverse Events version 4.0 (CTCAEv4U.S. Department of Health and Human Services, National Institutes of Health, National Cancer Institute, 2009).

Statistical evaluation was performed using Statistical Package for the Social Sciences (SPSS) version 25.0 (IBM Corporation, Armonk, NY), and scipy (1.6.3) and lifelines (0.26.0) python (3.7) packages (Python Software Foundation, Beaverton OR, United States).

## Results

### Patient and tumor characteristics

Patient characteristics are shown in Table 1. All patients had locally advanced and/or node positive disease. Ninety-three percent of the patients received iCT dominantly with Folfirinox. Seventy-three percent of the patients had primary disease, while the remaining had local recurrences after a Whipple operation. The mean CA19-9 value before SABR was 146 IU/mL on average.

**TABLE 1:** Patient and tumor characteristics.

No of Pts	15
Age, y, median (range)	67 (52–83)
Male/female	5/10
ECOG, median, (range)	1 (0–1)
Tumor size, mm, (range)	38 (25–60)
Primary	11
Recurrence (post-op)	4
T stage, *N*, (%)	
T3	1
T4	14
N+	3
Induction CT, (%)	14 (93)
Folfirinox/Gemcitabine (%)	10 (67)/4(33)
Regression/SD post-ICT	7 (50)/7 (50)
Number of CT cycles, median, (range)	7 (2–13)
Initial CA19-9, mean, U/mL (range)	308 (15–1,000)
Post-ICT CA19-9, mean, U/mL (range)	146 (0.6–395)

### Treatment characteristics/treatment time

Seventy-one percent (10/14) of the patients had titanium surgical clips, with an average number of 6 (1–10). One patient had lipiodol labelling, while three patients had bile duct stents. There were no intervention-related side effects.

ITV-based (5), phase-gated (5), or breath-hold (4) techniques were applied (Table 2). The median treatment time was 16.6 ± 11 min. On average, beams were interrupted once (range: 0–3) per treatment session. Treatment time and number of corrections were moderately correlated (*R* = 0.43, *p* < 0.01). The shortest treatment time was achieved by ITV-based treatment, however breath-hold-SABR required significantly less time compared to phase-gated treatment (18.8 ± 6.2 vs. 26.5 ± 13.4, *p* < 0.001).

**TABLE 2:** Characteristics of treatment verification, treatment time analysis.

Imaging for treatment planning	Dynamics/cine MRI	5/14
	4D CT, N/Σ	6/14
	4D CBCT, N/Σ	–
	DIBH CT	6/14
Intrafractional triggered kV imaging, N/Σ		14/14
Post-CBCT, N/Σ		12/14
Motion-management technique	ITV concept	5/14
	Respiratory gated	4/14
	DIBH technique	6/14
Total treatment time^a^, median ± SD, min	Σ	16.7 ± 10.8
	ITV concept (N:5)	12.1 ± 9.2
	Respiratory gated (N:5)	26.5 ± 13.4
	DIBH technique (N:5)	18.8 ± 6.2
Treatment delivery time^b^, median ± SD, min	Σ	7.3 ± 7
	ITV concept	3.8 ± 8
	Respiratory gated	10.5 ± 6.4
	DIBH technique	9.8 ± 4.3
Beam on time + triggering time, median ± SD, min	Σ	4 ± 2.5
	ITV concept	2.5 ± 0.5
	Respiratory gated	8.3 ± 1.1
	DIBH technique	5 ± 2
Time for 2D/3D ± other operational time, median ± SD, min	Σ	2.4 ± 6.1
	ITV concept	1.4 ± 7.9
	Respiratory gated	2.9 ± 5.8
	DIBH technique	4 ± 3.4
Correction time, median ± SD, min^c^	CBCT	4.9 ± 4
	Triggered imaging	1.5 ± 0.7
Number of corrections, mean (range)		1 (0–3)
Number of subjects, *N*		65
Imaging modality of corrections	CBCT, *N*	17/64
	Triggered imaging, *N*	48/65

^a^ Kruskal-Wallis H is 43.61 and is highly statistically significant with *p* < 0.001.

^b^ From the start of the first arc to the end of the second arc.

^c^ Mann-Whitney U is 15 and is highly statistically significant with *p* < 0.001.

**FIGURE 5:**
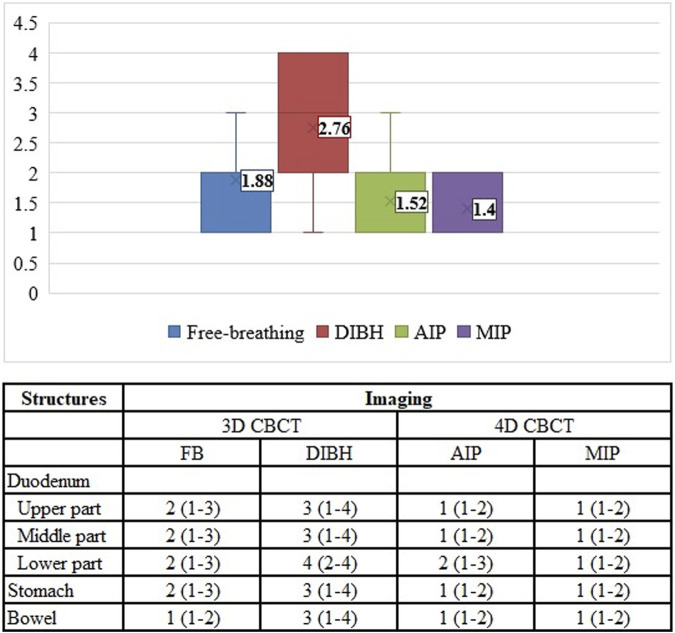
Image quality assessment of different CBCT modes. FB, free breathing; DIBH, deep inspiration breath hold; AIP, average intensity projection; MIP, maximum intensity projection.

There was a significant difference (*p* < 0.001) between the image quality of different CBCT modes. The best image quality was achieved by breath-hold CBCT (median: 3) compared to other modalities (DIBH vs. FB: 3 vs. 2, *p* = 0.002, DIBH vs. AIP: 3 vs. 1, *p* < 0.001, DIBH vs. MIP: 3 vs. 1, *p* < 0.001) (Figure 5). There was no significant difference (*p* > 0.05) between FB, AIP, and MIP CBCT. The global mean K-α value was 0.8 (97.5%), and for pre- and post-CBCT was 0.8 (97%) and 0.9 (98%), respectively, showing strong agreement among the five independent RTTs. The lowest agreement was achieved for the lower part of the duodenum (Table 3). There was no significant difference (*p* > 0.05) between pre-(full fan) and post (half fan) CBCT except for the middle part of the duodenum (*p* = 0.007).

**TABLE 3:** The degree of agreement between the RTTs’ evaluations.

	Organs at risk	Pre-CBCT			Post-CBCT		
		K-α	%	95% CI	K-α	%	95% CI
OAR position relative to PRV	Duodenum						
	Upper	1	100	1	1	100	1
	Middle	0.88	99	0.76–0.97	0.77	97	0.65–0.87
	Lower	0.78	94	0.68–0.88	0.71	94	0.59–0.82
	Stomach	0.89	99	0.82–0.96	0.86	98	0.75–0.95
	Bowel	0.82	98	0.69–0.93	0.75	96	0.63–0.86

Abbreviations: K-α, Krippendorff’s alpha; %, in % of Krippendorff’s alpha; 95% CI, the 95% confidence intervals; CBCT, cone beam computed tomography.

Dose-volume parameters are shown in Table 4. The average residual errors on pre-and post-breath-hold CBCTs were the following: vertical: 0 (0–0.34) cm, longitudinal: 0.2 (0–0.61) cm, lateral: 0.1 (0–0.37) cm. Consequently, the average (±SD) dose deviations from the original plan for D0.5cc of the duodenum, stomach, bowel, and high-dose PTV D2, D98 were −0.1% ± 1.2%, −1.1% ± 3.3%, 0.8% ± 3.3%, −1.6% ± 1.7%, and −0.3% ± 0.2%, respectively.

**TABLE 4:** Dosimetric results.

Target and OARs		5-fx SABR, (N:4)		5-fx SABR, (N:5)		5-fx SABR, (N:4)		15-fx DERT, (N:1)
		Mean	SD	Mean	SD	Mean	SD	Value
PTVlow	D98.00 [Gy]	28.3	6.2	25.4	2.8	31.0	3.5	37.6
	D2.00 [Gy]	33.4	5.1	37.4	2.6	45.7	4.8	56.7
PTVhigh	D98.00 [Gy]	31.0	4.7	32.7	4.4	40.3	3.0	57.4
	D2.00 [Gy]	33.4	5.2	37.7	2.6	46.1	4.9	60.1
Duodenum	D0.50cc [Gy]	15.8	4.8	27.8	5.5	32.1	0.6	44.3
Stomach	D0.50cc [Gy]	12.0	9.9	16.5	7.9	23.1	7.3	43.2
Bowel	D0.50cc [Gy]	23.5	8.5	23.5	8.5	23.5	8.5	43.2
Spinal Cord	D1.00cc [Gy]	8.0	2.2	8.0	2.2	8.0	2.2	19.2
Left Kidney	Mean dose	3.8	1.6	3.8	1.6	3.8	1.6	8.8
Right Kidney	Mean dose	4.4	1.8	4.4	1.8	4.4	1.8	8.1
Kidneys	Mean dose	4.0	1.3	4.0	1.3	4.0	1.3	8.4

In two patients (15 fraction DE-RT, and 5 fraction-SABR) offline treatment adaptations were made due to substantial volume and systemic positional changes of the stomach (Figure 4; Supplementary Figure S1). In both cases the stomach moved toward the high-dose area, violating the dose per fraction limit or the original PRV. Based on the CBCTs, delineations of the stomach’s new PRV-s were created, and re-optimization took place. One patient had a single treatment modification after the first four fractions, while in the second case two offline adaptations were required to fulfill the dose/fraction limit for the whole course of the treatment. In both cases only the adaptive plans were used for the remaining fractions. Dose-volume parameters per fraction for offline adaptive cases presented in Supplementary Figures S1–S3.

### Clinical outcome

**FIGURE 6:**
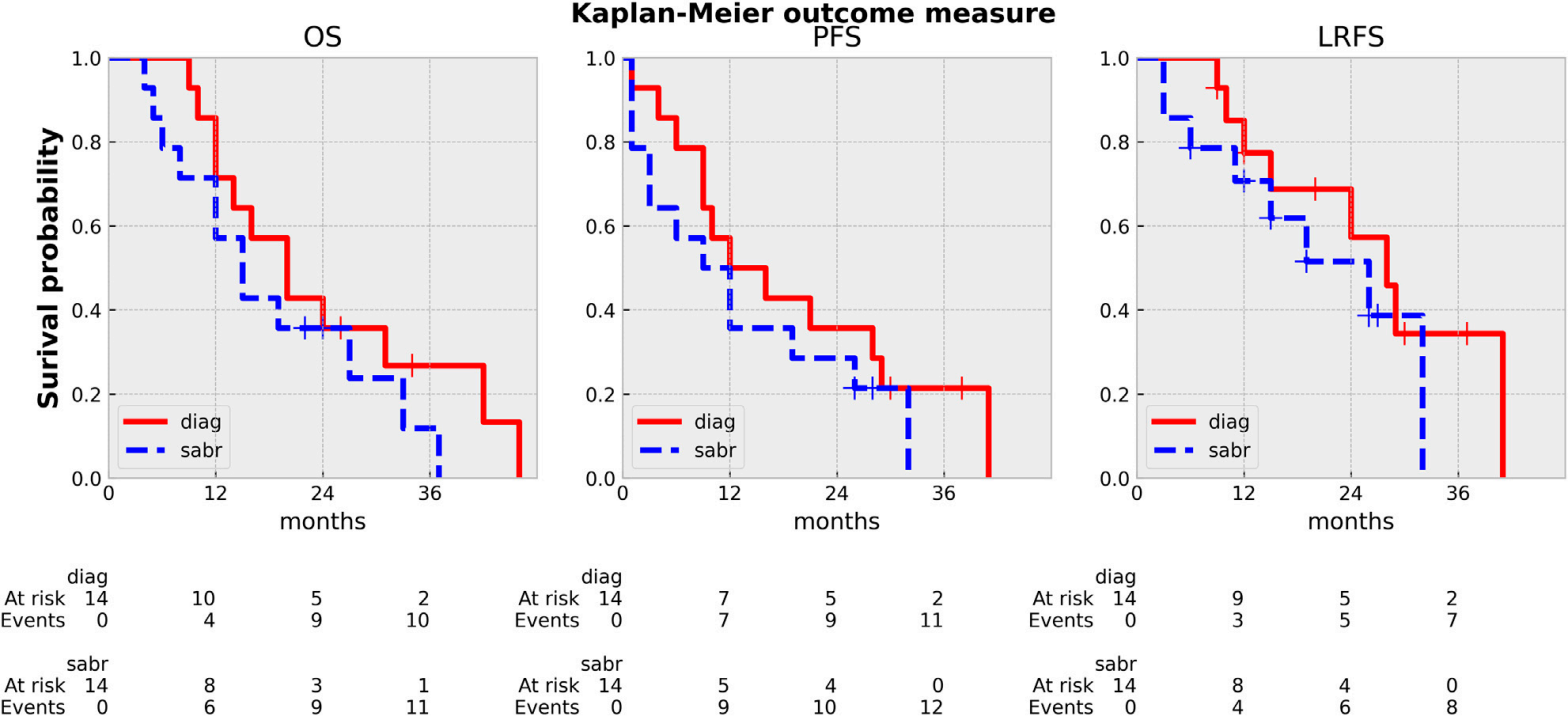
Kaplan Meier curves: OS (overall survival), PFS (progression free survival), LPFS (local-progression free survival). diag: from the start of induction CT, sabr: from the end of SABR.

Median OS and PFS_iCT_ was 20 (9–46) and 12 months (1–41), while OS/PFS_SABR_ was 15 (4–37) and 8 months (1–32), respectively (Figure 6). Five (36%) patients reached a minimum of 20 months survival [22–46 months (iCT), 22–37 months (SABR)]. The dominant pattern of failure (12/14) was distant metastasis (peritoneum: 12, liver: 3, lung: 3, pleura: 1). Eleven patients died of cancer, one patient died of cardiac decompensation during the re-induction of Folfirinox. The two adaptive cases are still alive after 28 and 36 months from the start of iCT without any sign of progression or toxicity. Eight patients (57%) recurred locally, half of them after 12 months, all but one with massive simultaneous systemic relapse. The 1-y LC_iCT/sabr_ was 78/70%, while the 2-y LC_iCT/sabr_ was 58/52% (Figure 6). The acute side effects were minimal with only Gr.1 nausea/vomiting/diarrhea/bloating in a single case with gastric abutment by the tumor (Figure 2). There was no late RT-related toxicity (diarrhea, GI bleeding/obstruction).

## Discussion

Our paper highlights an innovative Linac-based workflow for pancreatic SABR combining peritumoral fiducial-guided triggered imaging with gated or non-gated CBCT. Our ultimate goals were to develop full control of the entire workflow, improve time efficiency, and exploit the best CBCT image quality for better visualization of OARs and target with adaptation possibilities. From all these aspects we found that the breath-hold technique would be the first choice for pancreatic IGRT for SABR.

Pancreatic SABR requires the visualization of the tumor and OARs to increase dose delivery and maximize normal tissue protection. MRI-guided radiotherapy [14], with its excellent soft tissue contrast, combined with daily—even during fractional adaptation—real-time organ motion visualization, is already considered the treatment of choice for pancreatic SABR among all available technologies. As the incidence of LAPC increases, the need for pancreatic SABR is also growing. Unfortunately, MRI or CT-on-rail or CBCT based adaptive workflows are not widely available, mainly due to their elevated costs, reimbursement challenges and high resource requirements. The biggest disincentive from pancreatic SABR on conventional Linacs is the fear of side effects, which can be handled in two ways. Firstly, more careful patient selection compared to the advanced technologies and secondly, the development of a workflow that can address the above-mentioned challenges and ensures high dose delivery with minimized toxicity. Since direct visualization of OARS and tumor is not possible on Linac during treatment, we used fiducials as indirect surrogates, whose movement was controlled by kV images guided by a built-in software of the Linac. The rationale of using peritumoral fiducials was severalfold. Surgical exploration may detect peritoneal deposits, thus excluding these patients from further local treatment. It could also improve target definition on planning CT by incorporation of intraoperative findings, while allowing a multidimensional visualization of the target on triggered imaging, hence improving precision of our treatment (Figures 2, 3). Furthermore, the displacement of fiducials could also signal a positional and/or volumetric changes of duodenum/stomach on CBCT or on triggered imaging as they are inserted at the tumor/OAR interfaces (Figure 4). Each long-term survivor in our cohort had surgically implanted fiducials.

Treatment time is a crucial factor during SABR since prolonged radiation delivery is prone to have more errors. As expected, ITV-based treatment with abdominal compression was the most time efficient due to its lower complexity, mostly due to the benefit of non-moving targets such as retroperitoneal recurrences. Interestingly, breath-hold-SABR took only 18 min on average, 25% less than free breathing phase-gated treatment in addition to clearly better image quality. This can be explained by careful selection and training of breath-hold patients, as well as the time triggering feature of beam hold. During treatment, the Auto-beam hold function was switched off, so the radiation therapist was responsible for the beam control, which allows the number of breaths to be reduced. When the beam is held during time triggering, the system keeps shooting, providing a dynamic movement analysis just before or any time during radiation delivery. First, this fast confirmation of the fiducials could decide whether the breath-hold threshold should be fine-tuned or not. Second, the radiation therapist can give short verbal instructions (“breathe in or breathe out a little more/less”) to the patient, which permits continuous treatment and avoids unnecessary cessation of radiation delivery and consequently, the early exhaustion of the patient. Third, fiducial overlap or random displacement of the fiducials may occur, resulting in an undetectable or tilted position of the markers on triggered imaging, which would inevitably lead to an immediate interruption of radiation delivery if auto beam hold mode were active. It should be mentioned that during these treatments, no visual coaching device was available, so treatment time could be further decreased. Due to its complexity, considerably longer treatment time was reported with MR Linacs ranging between 83–90 min on average, decreasing toward 60 min with the introduction of audio feedback [26, 27]. However, it is counterbalanced by the intrafractional soft tissue visualization. Even with online adaptive CBCT based treatments, a total treatment time of 70 min was reported (18). CT equivalent CBCT embraced with ultrarapid image acquisition (6s) is coming up on the latest version of Halcyon.

**FIGURE 7:**
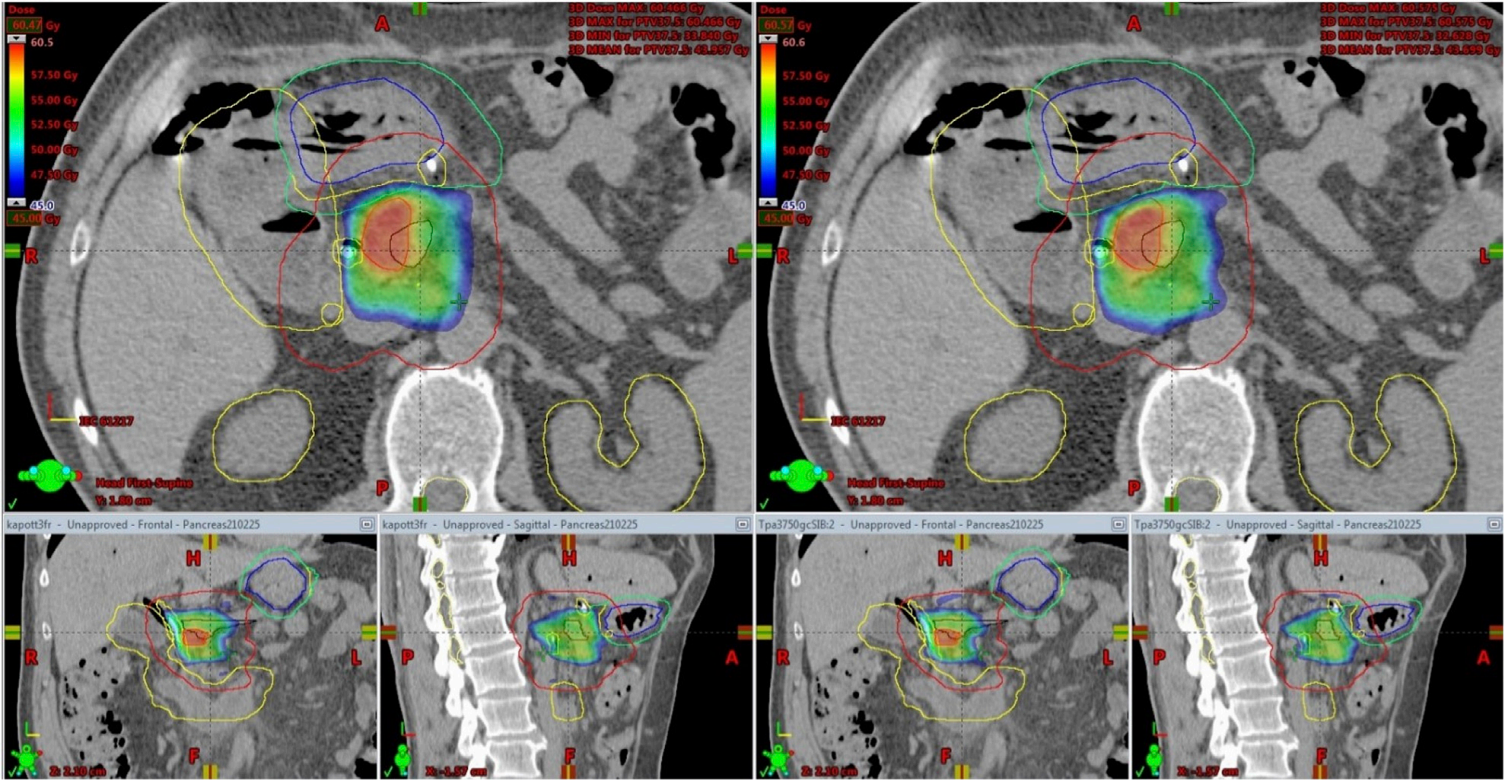
Offline treatment plan re-optimization based on CBCT contouring of the stomach (Blue: original stomach, yellow: original PRV, green: new PRV).

Again, CBCT may not be competitive with MRI, but tailored protocols can go a long way to improving image quality by reducing gas and internal motion artifacts and reducing treatment time. However, free breathing acquired images still suffer from respiration induced artefacts even with abdominal compression. In this aspect, breath-hold seems to be a good alternative. Moreover, reproducibility is not influenced by an abdominal compressor, which could cause even higher positional variations of the pancreas or OARs. In contrast, the technique needs careful patient selection and training. Our first experiences with breath-hold were very positive [22]. Instead of using only a descriptive 1–4-point scale to measure image quality, we sought to evaluate an offline treatment simulation of the visibility of OARs. Based on the opinion of five independent RTTs, breath-hold-CBCT can be used to reliably determine OAR position relative to PRV [K-α = 0.85 (0.71–1)]. Surprisingly, the agreement was the lowest for the lower part of the duodenum. The CBCT acquisition type was only an influencing factor for the mid-part of the duodenum, favoring the full arc mode. However, in the case of Spotlight mode, it is still above the minimum criteria value of K-α (0.66), and we consider that clinically acceptable. This means that breath-hold image quality is sufficient to monitor PRVs during treatment and capable of triggering offline treatment adaptation as we did in our two cases (Figure 4, 7). Moreover, Spotlight mode (Figure 4) reduces image acquisition time by half per CBCT, further reducing the number of breath-holds and the total treatment time.

Our clinical results (OS, PFS, LC) are comparable to those using the similar fractionation scheme [7]. In more than one-third of our cases, exceptionally good clinical outcomes were achieved including four patients with no evidence of progression >24 months (26–41) from the start date of ICT. Two of them are still alive without any sign of progression. In both adaptive cases (Figure 4, 7) there was a substantial response of the tumor and CA19-9 to polychemotherapy, and the prescribed dose was also the highest in these cases (Table 4). As expected, distant metastasis represented the dominant pattern of failure. It should be also mentioned that half of these patients had simultaneous local progression as well. We had only one patient with local-only recurrence as first failure. Recent studies demonstrated that dose escalation beyond a BED of 70 Gy was associated with improved OS [12, 14]. Moreover, BED higher than 100 Gy seems to further boost these clinical results [24, 28–30] beyond a median OS of 18 months, however prospective randomized trials are still warranted. In light of these results, the dose we prescribe can be considered conservative. We did not have either Grade 3 acute or late side effects which is impressive even with these conservative SABR doses. Notably, in trials using the same dose levels, Gr. 3 GI toxicity of 6%–10% was reported including Gr. 5 events as well [7, 13, 30]. We believe that our feasible toxicity profile is partly attributed to our advanced image verification workflow as well. Isotoxic high-dose SABR with improved or maintained serious side effects could be realized on MR-Linac. The SMART Pancreas study, applying 50Gy/5 BED100 Gy, met its primary objective with zero incidences of acute grade 3+ GI toxicity [14, 31, 32].

The limited number of cases, as well as heterogeneous, continuously evolving protocol, are potential weaknesses of our paper. On the other hand, on Linac, this advanced end-to-end workflow has never been presented and it is supported by comprehensive image quality and time efficiency assessments with the introduction of offline adaptive cases. We hope that our protocol could serve as an example to be followed by other Institutions with similar technologies.

In conclusion, Linac-based pancreatic SABR with triggered imaging and beam-hold is feasible. Peritumoral fiducials with time triggering improve utility and time efficiency. Breath-hold-CBCT provides the best image quality at a reasonable treatment time span with offline adaptation possibilities and should be considered as a first choice IGRT technique for pancreatic SABR. Clinical outcome is promising with negligible side effects. In selected cases, this workflow may be a viable alternative in clinics where MR-guided and/or online adaptive CBCT/CT technology is not available.

## Data Availability

The raw data supporting the conclusion of this article will be made available by the authors, without undue reservation.

## Supplementary material

SUPPLEMENTARY FIGURE S1 Offline adaptive cases. Case 1: 5 fraction SABR. Due to increased volume changes of the stomach during breath-hold **(A)** there was a clear trend of increased dose of the stomach on pre-and especially post-CBCT compared to the reference.

SUPPLEMENTARY FIGURE S2 Dose deviations of the Target (D2, D98) and Stomach (D0.5cc) from the original plan considering offline adaptations and residual errors. As a worst-case scenario (post-CBCT) a 5% increase in D0.5 cc of the stomach (from 32.1 to 33.9 Gy) and 8% decrease in D98 (from 34.95 to 32.08 Gy) could be estimated while still meeting hard constraints.

SUPPLEMENTARY FIGURE S3 Offline adaptive case 2: 15-fraction dose escalated treatment. Evolution of dose per fraction of OARs during the whole course of the treatment. After the 4th fraction, a single adaptation was applied and used consequently for the remaining fractions to avoid overtreatment of the gastro-duodenal junction.
