# Supplementary figures and images for: Pancreatic SABR using peritumoral fiducials, triggered imaging and breath-hold
Source: Pathol Oncol Res. 2023 Dec 21;29:1611456. doi: 10.3389/pore.2023.1611456 (PMC10767757; doi:10.3389/pore.2023.1611456)

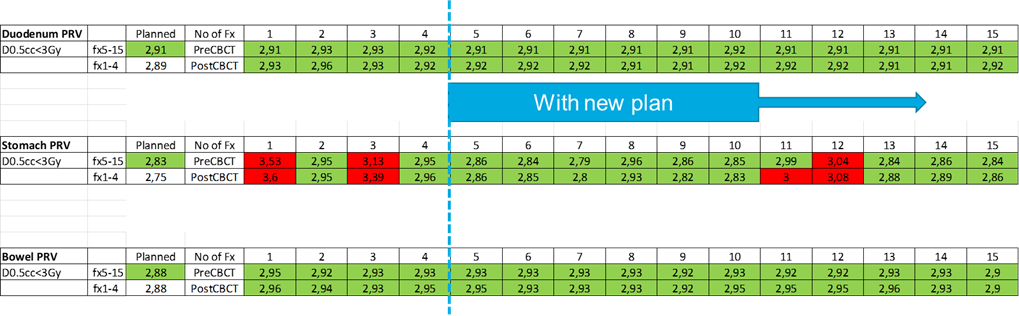

Supplement: Supplementary file 1 [file Image3.TIF]

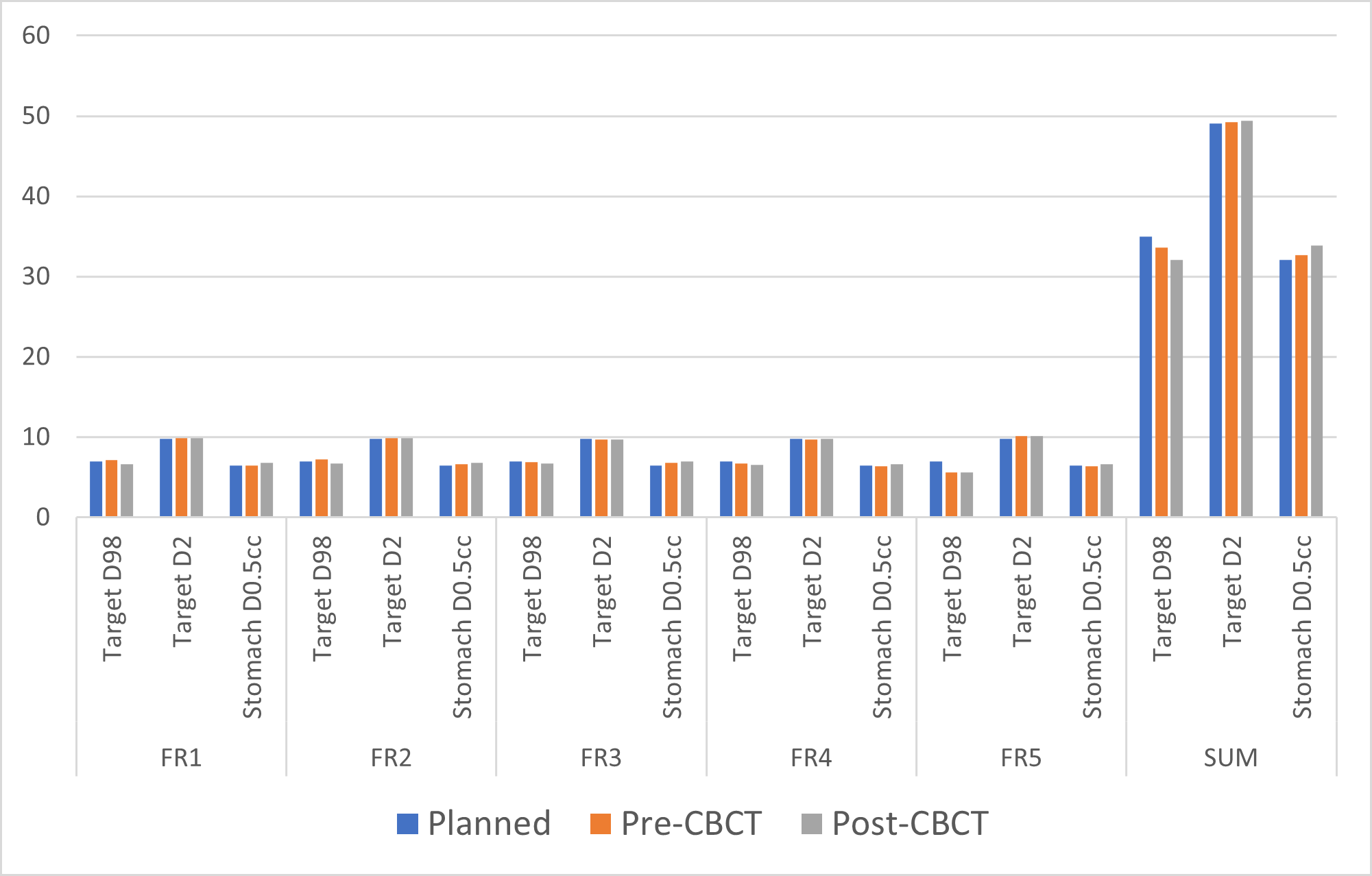

Supplement: Supplementary file 2 [file Image2.TIF]

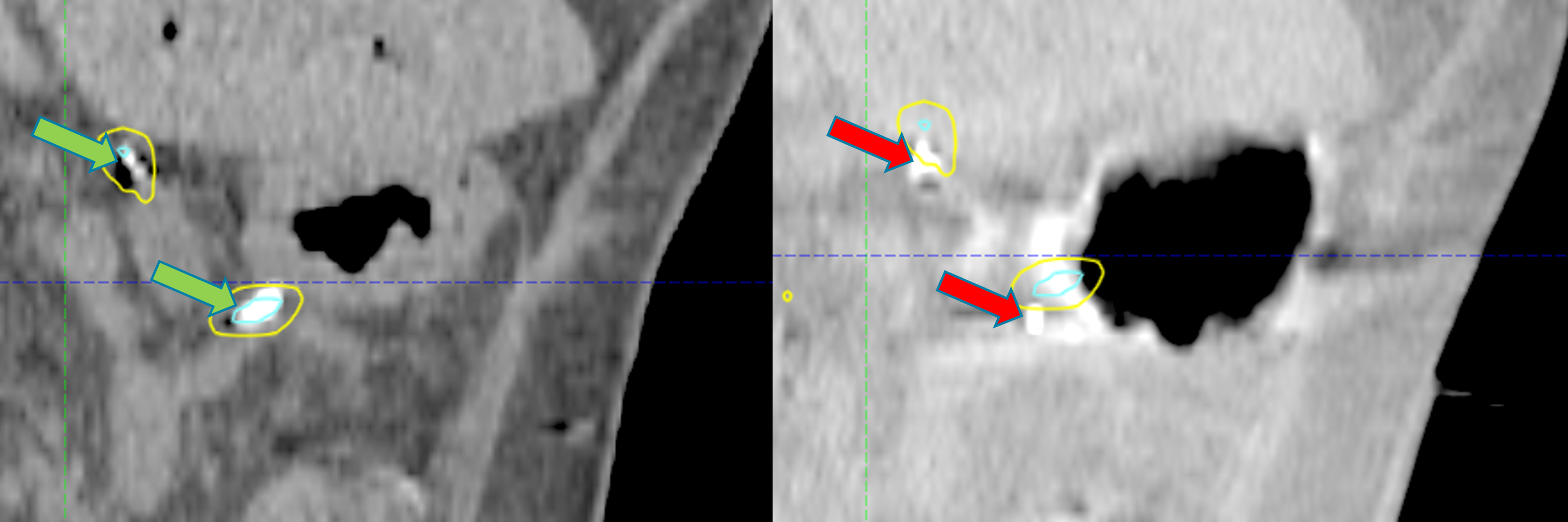

Supplement: Supplementary file 3 [file Image1.TIF]
